# Cavitation Erosion Prevention Using Laser Shock Peening: Development of a Predictive Evaluation System

**DOI:** 10.3390/ma16145096

**Published:** 2023-07-19

**Authors:** Wenlong Li, Hongbing Yao, Zhipeng Ding, Yuanhang Zhou, Pengyu Wei, Jiang Yue, Wei Su, Weihua Zhu

**Affiliations:** 1 College of Science, Hohai University, Nanjing 211100, China; 2 China Ship Scientific Research Center, Wuxi 214082, China

**Keywords:** laser shock peening, cavitation erosion, finite element analysis, micro-jet

## Abstract

Marine flow-passing components are susceptible to cavitation erosion (CE), and researchers have worked to find ways to reduce its effects. Laser Shock Peening (LSP), a material strengthening method, has been widely used in aerospace and other cutting-edge fields. In recent years, LSP has been used in cavitation resistance research. However, the current LSP research does not realize a comprehensive predictive assessment of the material’s CE resistance. This paper uses m stresses to develop a comprehensive set of strengthening effect prediction models from LSP to CE using finite element analysis (FEA). Results show that the LSP-1 sample (4 mm spot, 10 J energy) introduced a compressive residual stress value of 37.4 MPa, better than that of 16.6 MPa with the LSP-2 sample (6 mm spot, 10 J energy), which is generally consistent with the experimental findings; the model predicts a 16.35% improvement in the resistance of LSP-1 sample to water jet damage, which is comparable to the experimental result of 14.02%; additionally, interactions between micro-jets do not predominate the cavitation erosion process and the final CE effect of the material is mainly due to the accumulation of jet-material interaction.

## 1. Introduction

Marine resources are abundant and have garnered significant attention in contemporary oceanographic research [1,2]. The exploration and exploitation of these resources are crucial areas of focus [3]. High-speed, cross-flow components, such as propellers, tubes, and pumps, play a crucial role in marine resource exploration and exploitation equipment. However, these components are prone to damage from cavitation erosion over time, leading to a significant decrease in the equipment’s service life and reliability [4]. Copper alloys, stainless steel, and other materials with the properties of easy processing and strong resistance to cavitation erosion have been widely used in the marine industry [5]. Therefore, enhancing the CE resistance of copper alloys has been a critical research issue for researchers in various countries [6,7,8].

The treatments for cavitation erosion protection of existing materials are broadly divided into three categories: one is utilizing high-performance alloy materials through the manufacture of new materials or the alloy with trace elements to achieve high performance [9,10]; the second is setting high-performance coatings on the surface of the material, such as spraying organic or metallic coatings, to protect the internal metal [11,12]. Researchers have tried different kinds of high-performance alloys or metal coatings to improve CE resistance, and some remarkable results have been achieved, but it can still be further improved, especially in residual stress regulation [13]. Laser Shock Peening (LSP), an advanced technique widely used in aerospace and other critical military fields, has gained a lot of attention in the field of cavitation erosion resistance in recent years [14,15,16,17]. The principle of LSP technic is using laser beams to generate plasma shock waves to act on metallic materials to improve their fatigue and corrosion resistance [18,19,20,21,22]. S. Zabeen et al. used LSP to study the residual stress field around aero-engine specimens. The results show that the residual stress field is very stable in the fatigue test and can significantly improve the fatigue resistance of the material [23]. J. Chi et al. studied the microstructure and mechanical properties of titanium alloys before and after LSP treatment. The results show that LSP can significantly change the residual stress distribution, produce high-density dislocations, mechanical twins and grain refinement to improve its mechanical properties. [24]. Y. Bai et al. investigated the effect of nanosecond LSP on the life cycle performance of high-strength steels. The results show the presence of compressive residual stresses in the laser-treated area and a significant increase in microhardness, corrosion resistance, and fatigue life. [25]. C.Y. Wang et al. investigated the effect of LSP on the cavitation behavior of high-strength steel. The results show that the compressive residual stress can offset part of the fatigue load, effectively inhibit the emergence of cracks, and reduce the crack expansion rate, thereby improving the cavitation resistance of the material [26]. As a result, LSP can significantly improve material properties and improve the material’s CE resistance.

The investigation of LSP to enhance the resistance of materials to cavitation erosion has emerged as a prominent area of interest in contemporary research, encompassing both experimental and simulated approaches [26,27]. Most simulation studies on LSP have used finite element analysis (FEA). C.Y. Wang et al. conducted a simulation study of large-area LSP using FEA to investigate the effect of overlays on the CE of AISI 420 stainless steel [26]. Yang et al. studied the LSP-induced surface residual stress hole formation mechanism by analyzing the laser-induced shock waves generated using FEA [28]. Zhang et al. performed 3D FEA simulations of two-sided laser impact machining of 7075-T7351 alloy plates with a thickness of 2 mm to investigate the dynamic propagation and attenuation processes of shock waves within the material [29]. Numerical simulation studies on cavitation and cavitation erosion are conducted more using FEA, Computational Fluid Dynamics (CFD) and molecular dynamics simulation. Y. Tian et al. investigated the cavitation erosion behavior of ultrasonic sonar bars in large castings using numerical simulations and experiments on Al alloy cylindrical ingots [27]. Paul McGinn et al. proposed a new three-fluid volume framework, using CFD to analyze the behavior of cavitating sprays and other complex multi-fluid, multiphase fluid flows [30]. Asano Yuta et al. using a molecular dynamics simulation, investigated the effects of cavitation on the flow around a circular-cylinder array [31]. However, most studies on LSP against CE are limited to numerical simulations of LSP and cannot be connected with simulation studies of CE. The current study cannot establish evaluation systems from LSP to CE resistance improvement [8,32,33].

The dynamic CEL (coupled Eulerian-Lagrangian) analysis in ABAQUS is advantageous and widely used to simulate liquid-solid coupling [34,35,36]. The fundamental principle of CEL analysis is the Euler’s method, where the momentum and energy fluxes of the fluid are mapped in an Euler grid before the fluid starts to flow. The fixed Euler grid acts as a “detector” independent of space, monitoring the fluid motion in real time and accurately communicating the interaction between the fluid and the solid. In addition, the residual stress field model data obtained after the LSP simulation can be directly imported into the following CE simulation process, creating a bridge between the LSP simulation and the CE simulation. Therefore, this paper investigates the CE resistance of HSn70-1 after LSP by using CEL analysis method and provides predictions of the strengthening effect.

To address the above drawbacks, this paper proposes a comprehensive predictive evaluation model of the CE resistance effect of LSP based on FEA, which successfully uses residual stress as a bridge to realize the unification of LSP simulation analysis and material CE process simulation analysis, VDLOAD subroutines are rebuilt to reduce calculated amount. In this paper, the LSP process with two different parameters was simulated and analyzed, and the strengthening effect was initially evaluated in terms of compressive residual stress, surface morphology, and depth of the acting layer and compared with the experimental results. Subsequently, the data results obtained from the LSP simulation analysis were transferred to the CE simulation analysis using the residual stress as a bridge, and the results of the improved CE resistance of the material were successfully obtained. After that, the ultrasonic cavitation erosion experiments were compared and analyzed with the CE simulation results, and the final evaluation of the strengthening effect of the material in terms of accumulated mass loss, surface morphology, and surface roughness was performed. The model was further improved based on the experimental results. Finally, the micro-jet and material interaction mechanism during ultrasonic cavitation erosion is discussed.

## 2. Simulation and Experiment

To construct a comprehensive predictive evaluation system for integrating LSP and CE, this paper was divided into two parallel processes: simulation and experiment. The experiment and simulation were independent of each other, and there was no cross-use of data. In addition, this work selected data at two important nodes, LSP results and CE results, for side-by-side comparisons to validate the accuracy of the evaluation prediction system. The residual stresses were used as a bridge to connect the LSP model with the CE model. Simultaneously, the residual stresses on the surface of HSn70-1 samples treated with LSP were examined and the samples were used for CE experiments.

### 2.1. LSP Modeling and Experiment

#### 2.1.1. LSP Modeling

Considering the uniformity between simulation and experiment, a full-plate LSP model of an HSn70-1 sample with the size of 20 mm × 20 mm × 3 mm was established, the same size as the sample used in the subsequent experiment. The laser loading mode is also the same as the experiment. Two different processing parameters are used: LSP-1 and LSP-2, and the impact times are two, as shown in Table 1. The Johnson–Cook damage model, as demonstrated in Table 2, was added to the simulated models. A fine mesh of 10 μm was chosen to accurately simulate the evolution of the residual stresses in the material generated by LSP induction. The stress data is transferred from Explicit analysis to Standard analysis for overall dynamic stress balance after multiple impacts are completed to obtain a stable residual stress field.

#### 2.1.2. LSP Experiment

Tin brass is a great corrosion-resistant material both in freshwater and seawater, has good mechanical properties, and is widely used in parts of ships that come into contact with seawater. On the other hand, tin brass has better toughness, phase change is difficult under high-strain rates, and the dominant influence on the material properties is residual stresses during high-speed water jet erosion. For other materials, such as ceramic alloys and high-entropy alloys, it is the phase change rather than the residual stress that will play a dominant role in the material properties. Therefore, this work studied the cavitation resistance of tin brass (HSn70-1), a commonly used material in marine engineering, after laser shock peening. The specific chemical components are shown in Table 3. The tin brass sheet was cut into 20 mm × 20 mm × 3 mm specimens and the low temperature annealing of the material afterwards is designed to minimize the residual stresses introduced during the machining process. Resistance furnace will be used to slowly raise the specimen temperature to 300 °C, holding two hours, with the furnace cooling down to 150 °C below. This action was conducted to remove the introducing stresses during the machining process of the material and to ensure the accuracy of the residual stress results measured after the subsequent LSP machining. Surface treatment of the annealed samples was performed by sanding with #400 to #2000 sandpaper, followed by polishing with the diamond polish of 0.25 μm particle size. The polished samples were washed for 15 min using an ethanol solution and then kept in kerosene, waiting to be subjected to LSP.

**Figure 1:**
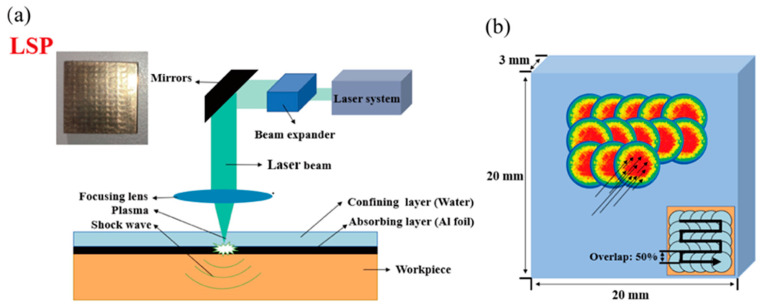
LSP simulation and experiments; (**a**) LSP experimental diagram; (**b**) finite element analysis model and laser loading method.

The LSP experiment uses the SGR-Extra-40-D laser (Beamtech Optronics Co., Ltd., Beijing, China, with a maximum single pulse energy of 40 J, a wavelength of 1064 nm, a frequency of 2 Hz, and a pulse width of 15 ns). Aluminum foil (thickness of 0.1 mm) was used as the energy absorbing layer, and incompressible water (about 1 mm thickness) as a confining layer. The schematic diagram of the experiment is shown in Figure 1a. Whole-surface LSP experiments were performed on all samples. The laser pulse energy was kept constant at 10 J. The spot diameters were set to 4 mm and 6 mm, respectively, which was to ensure the stability of the laser pulse energy. In addition, an s-shaped shock trajectory, 50% spot overlap, and two times impact were used. Laser related parameters are shown in Table 1. The laser loading method and path are shown in Figure 1b. To avoid randomness of experimental results, each group contained three specimens. To ensure the flatness of the sample surface, the surface of the reinforced and reference samples (the reference sample is abbreviated as the un-peened sample) was sanded with #800 to #2000 sandpaper.

### 2.2. CE Modeling and Experiment

#### 2.2.1. CE Modeling

**Figure 2:**
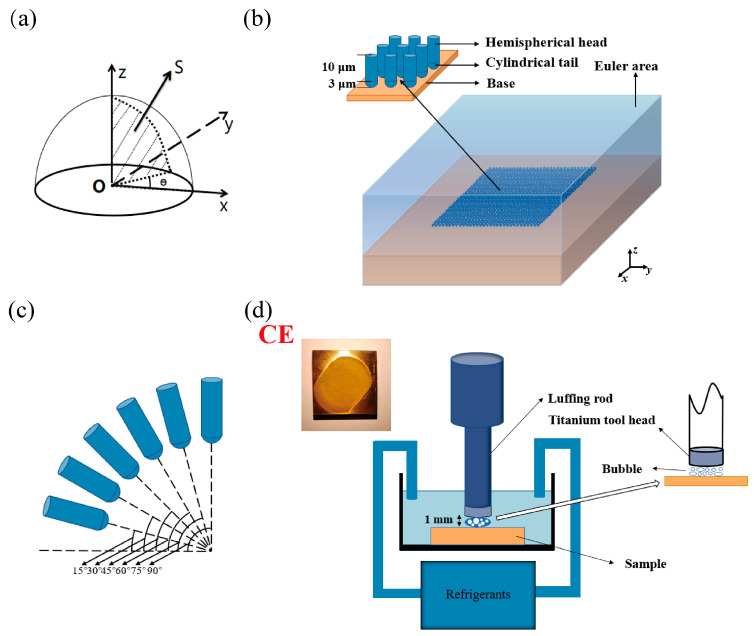
CE simulation and experiments (**a**) CE simulation integration schematic; (**b**) model description; (**c**) model incident angle description; (**d**) CE experimental diagram.

Considering the near-wall range situation, the micro-jets model was established to be better compatible with the experimental situation [37,38,39]. Several assumptions were made in the CE model: the subject of study is an ideal homogeneous material; the size and velocity of the jets generated by each collapse of the vacuole are equal; all jets are injected near the wall. The total effect *W* as the sum of the single jet effects *F*:

(1) dW=F(x,y,z)dxdydz

where *F* is a function about the spatial position, as shown in Figure 2a. Defining *S* as the sum of all single jet effects in the plane, then:

(2) dS=F(α)dα    α∈(0,90]

where *α* is the micro-jet incidence angle, and combining (1) and (2), then W∝S. According to the idea of differentiation, *S* can be regarded as the sum of an infinite number of independent variables. In this study, to reduce the computational effort, the value is taken at 15° intervals, Then, *S* is considered as the sum of the total effects of six different incidence angles.

(3) $$S=F(\frac{\pi }{2})+F(\frac{5\pi }{12})+F(\frac{\pi }{3})+F(\frac{\pi }{4})+F(\frac{\pi }{6})+F(\frac{\pi }{12})$$

According to Equation (3), the CE model of HSn70-1 was established. To ensure the model’s accuracy and reduce the calculated amount, microscopic cavitation modeling of the boundary with the infinite element was constructed. The initial model was set to 200 μm × 200 μm × 30 μm, and the grid size of the model is 1 μm. In addition, the initial model was improved through an analysis of the micro-jet’s action mechanism and a comparison of the experimental and simulation results. The final model after optimization was set to 70 μm × 70 μm × 30 μm, and the grid size of the model is 1 μm. The residual stress field model data obtained from the LSP simulation was imported into the CE model, and the coefficient of friction between the liquid-solid coupling was further added to get the CE model. Each water jet consists of a 3 μm diameter hemispherical head and a 10 μm long cylindrical tail, and the speed is 300 m/s, as shown in Figure 2b. The Euler area was set up to calculate the liquid-solid coupling. The Euler area must contain the entire array of jets and the sample model. The properties of water were given to the Euler area and the water jet array to simulate the whole model in a water environment. After preliminary simulations, the effect caused by the jet reached equilibrium at 3 μs, so a 5 μs analysis step length was set for displaying the analysis of the dynamics.

#### 2.2.2. CE Experiment

Both laser-impacted and un-peened samples were progressively polished to #2000 with different grits of sandpaper. Ultrasonic cavitation test equipment (Nanjing Xian’ou Instrument Manufacturing Co., Nanjing, China, XOQS-2500) with maximum ultrasonic power of 2500 W, ultrasonic frequency of 20 KHz, variable amplitude rod diameter of 15.9 mm, ultrasonic amplitude 0~100 μm was used for CE experiments. The ultrasonic experimental parameters: output energy 750 W, vibration frequency 20 KHz, amplitude 50 μm, distance of cavitation generator to material surface 1 mm, and maintained temperature of 22 °C, were set according to the ASTM G32 standard (Figure 2d). Deionized water was used as the medium for this experiment. Each sample in the CE device was removed every 30 min, ultrasonically cleaned with an aqueous alcohol solution for 10 min, and dried in a blast drying oven for 20 min. Samples were weighed using a 1000 ppm balance after cooling, and each time, the mass loss of the samples was noted. The total time of cavitation of tin brass samples was 5 h.

## 3. Results and Discussion

The LSP simulation was used to compare with the experimental results to evaluate and select the strengthening effect of the LSP samples; after that, the CE model was modeled and verified by exploring the interaction law between the cavitation micro-jet and the material in the near-wall condition; finally, the CE behavior and results of the samples were used to provide a final evaluation of the CE resistance of the samples and to verify the model results.

### 3.1. Comparison of LSP Simulation and Experimental Results

**Figure 3:**
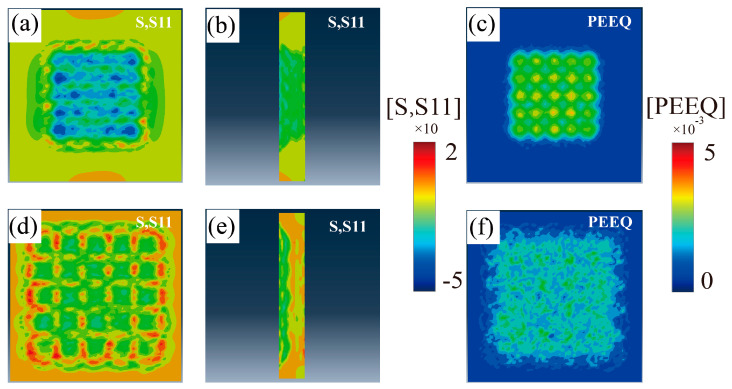
Comparison of LSP simulation results (**a**,**b**) first principal stress results for LSP-1; (**c**) PEEQ results for LSP-1; (**d**,**e**) first principal stress results for LSP-2; (**f**) PEEQ results for LSP-2.

**Figure 4:**
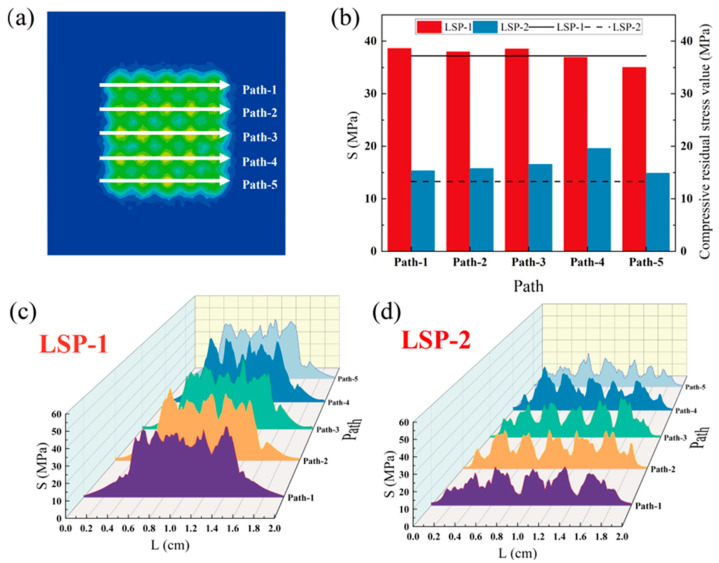
Comparison of LSP simulation results with experimental results (**a**) extraction path diagram; (**b**) comparison of LSP simulation and experimental residual stress values on the path (the bar is the simulation results; the line is the experimental results); (**c**) and (**d**) S-value on five paths for LSP.

The simulation results of the first principal stress (S11) are shown in Figure 3a,d, where the blue part indicates the presence of compressive residual stress, and the red and yellow areas appearing in the graph indicate the presence of tensile residual stresses. The overall stress distribution of models after LSP-1 and LSP-2 treatments is approximately the same, concentrated near the spot area’s center. In addition, the equivalent plastic strain (PEEQ) results after strengthening with two LSP parameters are shown in Figure 3c,f. The surface morphology of the model processed with the LSP-1 parameter has a significant regularity with a considerable point of deformation at the central area of the laser spot, while the surface morphology of the sample processed with the LSP-2 parameter is dispersed. It is because LSP-2 has smaller laser spot energy density than LSP-1, and with the same single pulse energy, LSP-1 has a smaller spot and more concentrated energy, while LSP-2 has a larger spot and more dispersed energy. It is clear that the enhancement effect of LSP-1 is significantly better than that of LSP-2. To verify the accuracy of the simulation results, five paths (Path 1–5) in the S (the von Mises Stress) result data were taken as in Figure 4a to measure and count the simulation results for LSP-1 and LSP-2 and further compare the simulation results with the experimental results.

The compressive residual stress values for the straight lines on the five paths (Path 1–5) in LSP-1 and LSP-2 models are compared, as shown in Figure 4c,d. The results show that LSP-1 and LSP-2 showed a similar trend in the compressive residual stress values for the different paths. The compressive residual stresses achieved through the LSP-1 machining parameters exhibit greater effectiveness, uniformity, and reduced undulation in comparison to those obtained through the LSP-2 machining parameters. Figure 4b shows the average compressive residual stress values for the five paths in the LSP-1 and LSP-2 models. The trend is the same for either loading parameter under Path 1–5, and the maximum residual stress value exhibited by the sample under the LSP-1 loading parameter is comparatively more substantial than that under LSP-2. After testing the residual stresses in the sample after LSP, the measured compressive residual stress values are 37.2 MPa for LSP-1 and 13.3 MPa for LSP-2, comparable to the simulated values of 37.4 MPa and 16.6 MPa. The surface morphology and compressive residual stress values of all samples are correctly predicted by the model.

In summary, the samples under LSP-1 processing parameters have better strengthening effects regarding strengthening depth and compressive residual stress values, so the LSP-1 samples and models were used next for CE experiments and simulations. In addition, it can be clearly seen that the residual stress values between experiment and simulation are different, and the inherent error in experimental testing may be the main reason for this difference. The simulation responds well to the law of stress change on the surface and inside the model, and it predicts accurate residual stress values. Due to the limitations of experimental testing, only a restricted number of detected values can be collected. As a result, there may be a slight difference between the predicted values and the detected values.

### 3.2. CE Simulation and Experimental Results

#### 3.2.1. CE Simulation Results

**Figure 5:**
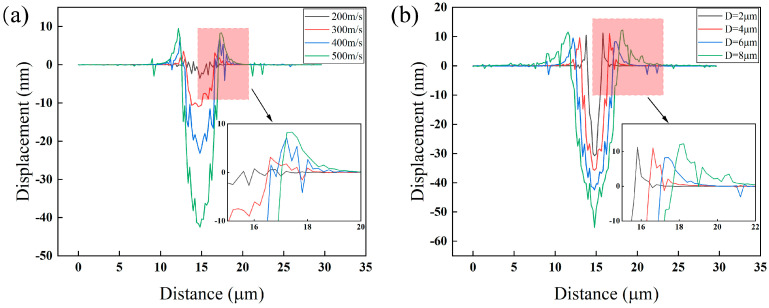
The law of interaction between single jet and material (**a**) effect of different jet velocities; (**b**) Effect of different jet sizes.

In the near-wall region, the breakdown of bubbles produces micro-jets, resulting in CE. Therefore, it is important to conduct pre-simulations to investigate the connection between micro-jet properties, such as dimensions and velocity, and material damage. To enhance the obviousness of the pre-simulation outcomes, a jet having a length of 14 μm and a diameter of 6 μm was utilized. Figure 5 shows the connection between the properties of individual micro-jet and the deformation displacement of the material surface. Figure 5a: 200, 300, 400, and 500 m/s micro-jets produced craters, with similar diameters, and different depths of 3.6, 10.9, 23.2, and 42.5 nm, respectively. The results indicate that, assuming a constant jet size, the magnitude of surface deformation resulting from the jet increases significantly as the jet velocity rises, while the scope of the crater remains relatively stable. Figure 5b shows the relationship between the jet diameter and the material surface displacement for a jet velocity fixed at 500 m/s: with the increase of the jet diameter, the larger the crater diameter, but the depth change is not significant. On the other hand, by observing the bulge around the crater in Figure 5, it is found that the crater bulge is higher and more extensive as the velocity increased; with the increase of the jet diameter, the crater bulge height did not vary considerably, but the bulge scope changed very significantly. The higher velocity or larger size of the micro-jet represents more energy carried, a stronger stress wave generated, and a larger area of influence. The micro-jet compresses the material in the center of the action area to form a plastic deformation, part of material escapes to the surrounding area and eventually causes a bulge at the edge of the action area. The greater the degree of these bulges on the material surface, indicates greater plastic strain at the point and greater vulnerability to damage during the ensuing CE process. The result of the interaction between the micro-jet and the material is primarily impacted by the velocity and size of the micro-jet. The depth of crater and height of bulge are mainly influenced by the velocity of the jet, while the range of crater and the scope of bulge are primarily affected by the size of the jet. Thus, considering the law of jet action and the calculated amount, a micro-jet with a velocity of 300 m/s consisting of a hemispherical head of 3 μm in diameter and a cylindrical tail of 10 μm was finally used in this paper to investigate the CE resistance of the material after LSP.

**Figure 6:**
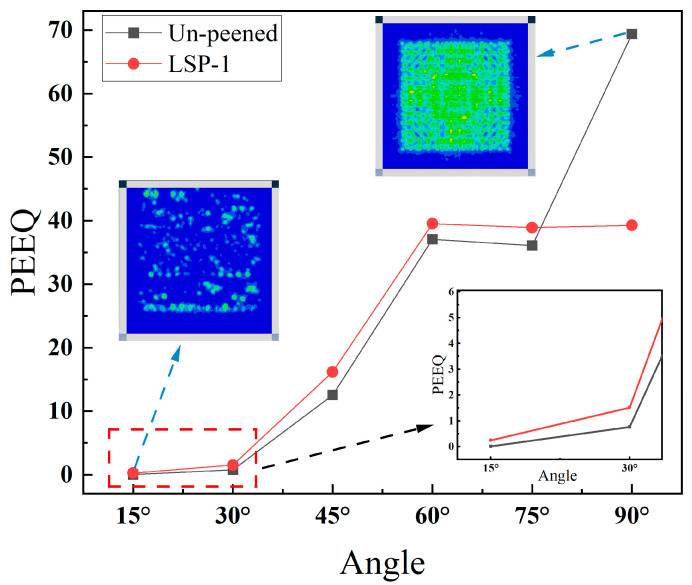
PEEQ results for different incidence angles.

The tearing and squeezing of the material caused by stress will result in material damage, therefore the equivalent plastic strain (PEEQ) of the model can be counted to estimate the model’s damage [40,41]. Figure 6 shows the PEEQ results for different incidence angles. It is obvious that the smaller the angle of incidence, i.e., the closer the jet direction is to the model’s surface, the more minor the damage of the jet on the model. The degree of damage gradually decreases as the angle decreases, and the incident angle of 90° shows the maximum equivalent plastic strain for both the LSP-1 model and the un-peened model. The reason for this phenomenon could be that the smaller the angle of incidence, the larger the contact area between the jet and the model will be. The smaller the angle of incidence, the more energy is consumed in friction, and the less energy is transferred to the model’s interior. It is noteworthy that the LSP strengthening manifests itself in a significant reduction of the equivalent plastic strain only at a 90° incidence angle. At the same time, there is no strengthening effect for other incidence angles, which is contrary to our prediction of introducing a residual stress field globally. The prediction of the overall reinforcement effect was calculated to be 13.01%. In this simulation, priority was given to the fact that more water jets should be acting on the model surface, and the interaction between the jets was neglected. However, the interaction between jets is also an integral part of the CE process, so discussing the effect of jet spacing on the model is critical. The effects of jet spacing on model results will be discussed in the following paragraphs.

**Figure 7:**
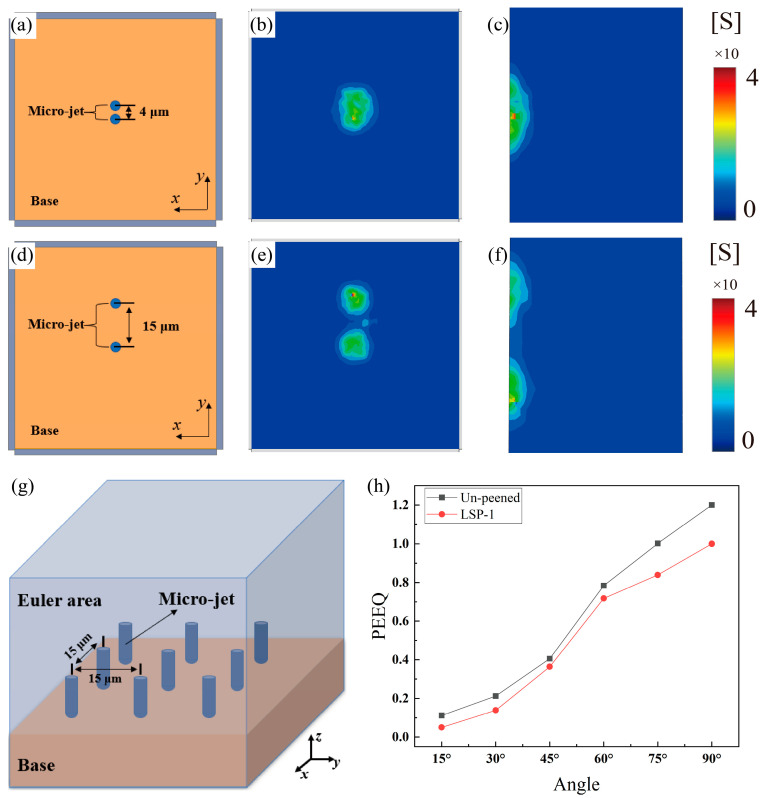
Connection between CE simulation and injection distance (**a**–**c**) results for the jet spacing of 4 μm; (**d**–**f**) results for the jet spacing of 15 μm; (**g**) the small model assembly schematic; (**h**) the small model results.

In the initial model, the diameter of the jets was 3 μm, and the distance between the jets was 4 μm. The pre-model shows that the stress range of the single jet on the model at equilibrium is about 7.5 μm, which is 2.5 times the jet diameter, so two micro-jets with 4 μm and 15 μm spacing are set to investigate the effect of inter-jet interaction on the model. As shown in Figure 7a–f, the stress behavior under the action of two jets with different micro-jet spacing proves that, for the 4-μm spacing jet, the effects of the two micro-jets are coupled with each other, making the effect of action larger. While for the 15-μm spacing jets, the two micro-jets act independently, the effect of mutual coupling between the jets is reduced, and the effect of action is smaller. The calculated total impact of the action at a jet spacing of 4 μm is 0.1927, approximately 20% higher than that of 0.1556 at 15 μm, as shown in Table 4. The results are different for the two jet spacings because a bigger spacing lead the two stress waves to weaken before interacting, causing less damage. Therefore, the determination of the jet spacing is crucial to constructing more accurate models.

To ensure the accuracy of the model, some phenomena in the experiment are considered. The type of experimental apparatus used in the CE experiments is an ultrasonic cavitation apparatus, based on the principle that ultrasonic pulses produce a pressure drop in water; thus, cavitation occurs, resulting in CE to the material [42]. In the experiment, it was found that the size scales of bubbles caused by ultrasonic cavitation are very different, and the giant bubbles escaping to the outside have prominent time intervals, so while there may be some interaction between the jets, the direct interaction between the independence micro-jet and the sample should dominate. Therefore, based on the above results, a more accurate and less computationally intensive small CE model (Figure 7g) of 9 micro-jets with a jet spacing of 15 μm was developed. As Figure 7g shows the small model assembly schematic diagram, Figure 7h shows the equivalent plastic strain results of the un-peened and LSP-1 models at different incidence angles. The trend of PEEQ results for the small model is approximately the same as that of the large model. The small model shows that the effect of reinforcement is reflected in all directions. The prediction of the overall reinforcement effect was calculated to be 16.35%.

#### 3.2.2. CE Experimental Results

**Figure 8:**
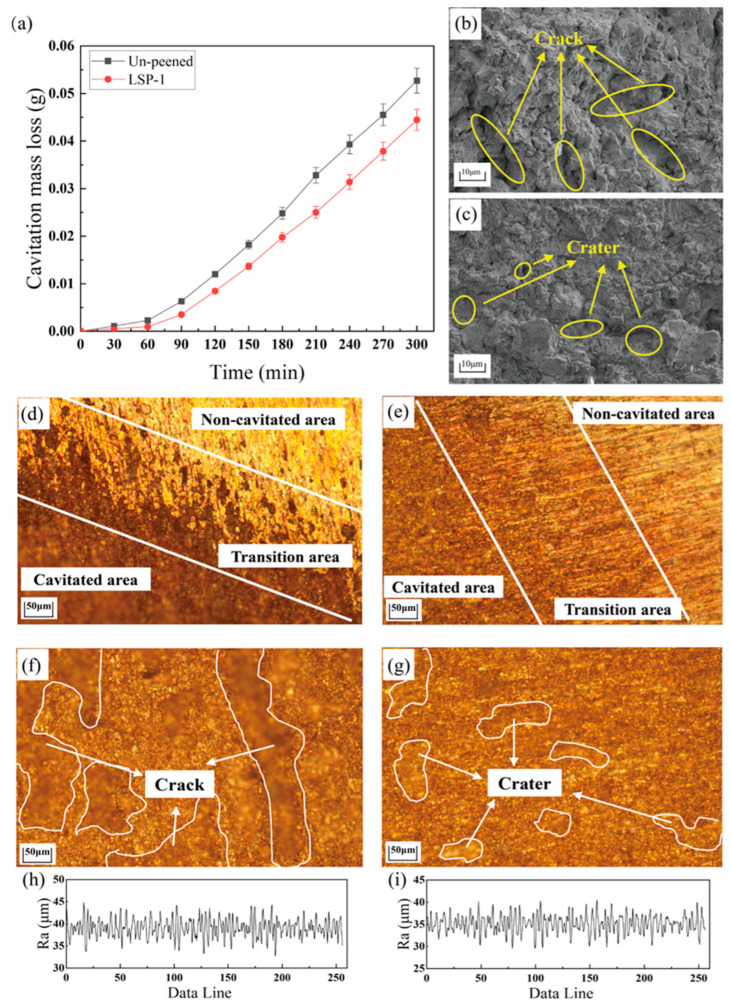
CE experimental results (**a**) CE mass loss curve; (**b**) SEM image of the un-peened sample; (**c**) SEM image of the LSP-1 sample; (**d**) and (**f**) the un-peened sample optic microscope; (**e**) and (**g**) the LSP-1 sample optic microscope; (**h**) the un-peened sample line roughness; (**i**) the LSP-1 sample line roughness.

To further verify the accuracy of the model, ultrasonic cavitation experiments were carried out, and the degree of damage to the material is estimated by using the ultrasonic cavitation mass loss method [43,44]. Figure 8a shows the un-peened sample and the LSP-1 sample’s ultrasonic cavitation mass loss curves. The mass loss curves of both samples have the same trend, but the incubation period time of the LSP-1 sample increases. The weight of all samples slightly decreases during the incubation period, and at the end of the incubation period, the material first flakes off at material defects and grain boundary slips, then pits and cracks are formed and finally cause a large area of the material surface to fall off [45,46,47]. The compressive residual stresses introduced by LSP prevent the expansion of these cracks, and then the CE resistance of the material is improved. The total mass reduction of the LSP-1 sample was 44.5 mg at 300 min of cavitation time, which was 15.66% less than the 52.7 mg of the un-peened sample, and the loss rate during the stabilization period was 0.22 mg/min less than the 0.24 mg/min of the un-peened sample.

The CE process of two samples is shown in Figure 8. The experimental area of the un-peened sample has a clear demarcation line with the non-experimental area, and the height difference of the sample surface is significant. In contrast, the transition area of the LSP-1 sample is relatively smooth and has apparent resistance to the CE. In addition, the un-peened sample has many cracks in the central region of CE, and the damage is obvious. The LSP-1 sample has only a few round crater damages in the central region of CE, which indicate that LSP can resist the plastic strain caused by CE and verify the previous simulation’s results. And then, the surface roughness of the sample after CE is further investigated; the line roughness of the un-peened sample ranged from 35 to 45 μm, while the overall line roughness of the LSP-1 sample was 30 to 40 μm. The average roughness of the un-peened and LSP-1 samples was 1.62 μm and 1.58 μm, respectively, which indicated that the LSP-1 samples have a smoother surface and are more resistant to CE. It has been pointed out that the destruction of micro-jets at grain boundaries is caused by tensile residual stresses [48]. Tensile residual stresses increase the likelihood of cracks and reduce the ability to resist plastic deformation, making microcracks easier to extend along grain boundaries [26]. In addition, the compressive residual stress can reduce some of the fatigue load and inhibit fatigue crack formation. As a result, the compressive residual stress reduces fatigue crack expansion, crater expansion, and further material spalling, and mass loss in LSP-1 specimens, smaller than in un-peened samples. The overall strengthening effect of the LSP-1 sample was calculated to be 14.02%, which is comparable to the model prediction of 16.35% and slightly less than the simulation result. Despite the fact that the predictive model correctly predicts the cavitation resistance of LSP-1 specimens, there is still an error of about 2% between the experimental results and the prediction model results. The probable reason for that is: 1. Boundary conditions, where the infinite element is set at the model borders, have been frequently employed in LSP simulation studies to prove their viability [22,26]. But the infinite element performance in CE simulation, a new finite element study field, is unclear. 2. Despite the strict temperature control method in accordance with ASTM G32 being adopted, the temperature of the liquid in the vessel still showed a significant increase during CE experiment period. This increase in temperature probably had a detrimental effect on the metal material, making it more vulnerable to damage. 3. The increase in surface roughness during the CE process is a possible reason for the decrease in the material’s resistance to CE. Nevertheless, the prediction results of the small model are approximately consistent with the experimental results, indicating that the interaction between micro-jets does not dominate in the CE experiments.

## 4. Conclusions

This study presents a novel approach to developing a comprehensive prediction model that utilizes laser shock peening (LSP) to enhance the cavitation resistance of materials. The model utilizes residual stress as a bridge and effectively integrates the LSP model with the CE micro-jet model, which is validated through experimentation. This approach effectively addresses the issue of isolation in the conventional simulation analysis of LSP and CE. The simulation and experimental results of the LSP part show that LSP-1 models and samples are obtained with a more uniform strengthening effect, greater depth of action, and better regularity of surface morphology than LSP-2. The average compressive residual stress values of 37.4 MPa and 16.6 MPa in simulation were obtained for the two processing parameters, which were generally consistent with experimental test results of 37.2 MPa and 13.3 MPa, and the preliminary judgment of CE resistance for both parameters was successfully made. The simulation and experimental results of the CE part that adopted LSP-1 as further research objects show that the LSP-1 sample showed modified surface roughness, surface morphology, and CE resistance. The final enhancement value of CE resistance predicted by the model is 16.35%, comparable to the experimentally obtained value of 14.02%, demonstrating that the CE model makes accurate predictions for the experiment. In addition, by exploring the pattern of micro-jets and material CE damage behavior in the near-wall range, it was found that the depth of crater and height of bulge are mainly influenced by the velocity of the jet, while the range of crater and the scope of bulge are primarily affected by the size of the jet. The simulations and experiments also show that a larger jet spacing attributes to a more accurate and realistic model, further indicating that microscopic inter-jet interactions do not dominate the overall CE experiments and that the final cavitation effect is the result of cumulative single-jet-material interactions. Although the model proposed in this research gained accuracy in the prediction of experiments, this research still has some limitations: 1. The cavitation mechanism is complex; thus, this article investigates interactions between the micro-jet and material in the near-wall range. 2. Inherent experimental errors, for example, scrapes while weighing, cleaning, drying, and other experimental operations, may affect the results. In further research, the micro-jet action law during CE process will be continuously improved, for example, the material surface roughness, the complex fluid behavior of micro-jets and other factors will be considered. It should be mentioned that this paper provides a research basis to further elucidate the CE process’s mechanism and the prediction of the LSP reinforcement effect, which is expected to offer guidance to the marine corrosion protection industry.

## Figures and Tables

**Table 1:** Comparison of two laser parameters.

		Parameter	Laser Energy (J)	Impact Times	Overlap Rate	Spot Diameter (mm)	Power Density (GW/cm^2^)
Sample							
LSP-1			10	2	50%	4	7.96
LSP-2			10	2	50%	6	3.54

**Table 2:** The Johnson-Cook damage model parameters.

Parameter	ρ (g/cm^3^)	A (MPa)	B (MPa)	C	n	m
Value	8.41	190	495.4	0.0021	0.54	1.45

**Table 3:** HSn70-1 chemical components.

Elements	Cu	Sn	Pb	P	Fe	As	Zn
Percentage (wt%)	59.0~61.0	0.8~1.3	0.05	0.01	0.10	0.03~0.06	Balance

**Table 4:** Comparison of stress results for two different jet distances.

Distance	4 μm	15 μm
S (μm^2^)	165.68	154.04
H (μm)	3.66	3.15
PEEQ	0.1927	0.1556

## Data Availability

Not applicable.

## References

[1] Bax N., Novaglio C., Maxwell K.H., Meyers K., McCann J., Jennings S., Frusher S., Fulton E.A., Nursey-Bray M., Fischer M. (2022). Ocean Resource Use: Building the Coastal Blue Economy. Rev. Fish Biol. Fish..

[2] Kappenthuler S., Seeger S. (2021). Holistic Evaluation of the Suitability of Metal Alloys for Sustainable Marine Construction from a Technical, Economic and Availability Perspective. Ocean Eng..

[3] Dance A. (2020). Ocean Explorer. Nature.

[4] Park I.C., Han M.S., Kim S.J. (2019). Cavitation Damage Characteristics of Shot Peened ALBC3 Alloy. J. Nanosci. Nanotechnol..

[5] Luo Q., Zhang Q., Qin Z., Wu Z., Shen B., Liu L., Hu W. (2018). The Synergistic Effect of Cavitation Erosion and Corrosion of Nickel-Aluminum Copper Surface Layer on Nickel-Aluminum Bronze Alloy. J. Alloys Compd..

[6] Rakhmonov J.U., Bahl S., Shyam A., Dunand D.C. (2022). Cavitation-Resistant Intergranular Precipitates Enhance Creep Performance of θ′-Strengthened Al-Cu Based Alloys. Acta Mater..

[7] Park S.H., Phan T.H., Park W.G. (2022). Numerical Investigation of Laser-Induced Cavitation Bubble Dynamics near a Rigid Surface Based on Three-Dimensional Fully Compressible Model. Int. J. Heat Mass Transf..

[8] Sreedhar B.K., Albert S.K., Pandit A.B. (2017). Cavitation Damage: Theory and Measurements—A Review. Wear.

[9] Chen F., Du J., Zhou S. (2020). Cavitation Erosion Behaviour of Incoloy Alloy 865 in NaCl Solution Using Ultrasonic Vibration. J. Alloys Compd..

[10] Siemek K., Eseev M.K., Horodek P., Kobets A.G., Kuziv I.V. (2021). Defects Studies of Nickel Aluminum Bronze Subjected to Cavitation. Appl. Surf. Sci..

[11] Ribu D.C., Rajesh R., Thirumalaikumarasamy D., Kaladgi A.R., Saleel C.A., Nisar K.S., Shaik S., Afzal A. (2022). Experimental Investigation of Erosion Corrosion Performance and Slurry Erosion Mechanism of HVOF Sprayed WC-10Co Coatings Using Design of Experiment Approach. J. Mater. Res. Technol..

[12] Dinu M., Wang K., Mouele E.S.M., Parau A.C., Vladescu (Dragomir) A., Liang X., Braic V., Petrik L.F., Braic M. (2023). Effects of Film Thickness of ALD-Deposited Al_2_O_3_, ZrO_2_ and HfO_2_ Nano-Layers on the Corrosion Resistance of Ti(N,O)-Coated Stainless Steel. Materials.

[13] Chen W., Voisin T., Zhang Y., Forien J.B., Spadaccini C.M., McDowell D.L., Zhu T., Wang Y.M. (2019). Microscale Residual Stresses in Additively Manufactured Stainless Steel. Nat. Commun..

[14] Wang H., Huang Y., Zhang W., Ostendorf A. (2018). Investigation of Multiple Laser Shock Peening on the Mechanical Property and Corrosion Resistance of Shipbuilding 5083Al Alloy under a Simulated Seawater Environment: Publisher’s Note. Appl. Opt..

[15] Soyama H., Kuji C. (2022). Improving Effects of Cavitation Peening, using a Pulsed Laser or a Cavitating Jet, and Shot Peening on the Fatigue Properties of Additively Manufactured Titanium Alloy Ti6Al4V. Surf. Coat. Technol..

[16] Maleki E., Bagherifard S., Unal O., Jam A., Shao S., Guagliano M., Shamsaei N. (2023). Superior Effects of Hybrid Laser Shock Peening and Ultrasonic Nanocrystalline Surface Modification on Fatigue Behavior of Additive Manufactured AlSi10Mg. Surf. Coat. Technol..

[17] Feng A., Wei Y., Liu B., Chen C., Pan X., Xue J. (2022). Microstructure and Mechanical Properties of Composite Strengthened High-Chromium Cast Iron by Laser Quenching and Laser Shock Peening. J. Mater. Res. Technol..

[18] Liu L., Wang J., Zhou J. (2019). Characterization and Analysis on Micro-Hardness and Microstructure Evolution of Brass Subjected to Laser Shock Peening. Opt. Laser Technol..

[19] Li J., Chen S., Zhu W., Zhao Y., Liu L., Wang Z., Pan H. (2023). Microstructural Response and Surface Mechanical Properties of TC6 Titanium Alloy Subjected to Laser Peening with Different Laser Energy. Opt. Laser Technol..

[20] Gu J., Luo C., Ma P., Xu X., Wu Y., Ren X. (2021). Study on Processing and Strengthening Mechanisms of Mild Steel Subjected to Laser Cavitation Peening. Appl. Surf. Sci..

[21] Sun Y., Wu H., Du H., Yao Z. (2022). Investigation of Strain Fatigue Behavior for Inconel 625 with Laser Shock Peening. Materials.

[22] Wang M., Wang C., Tao X., Zhou Y. (2022). Numerical Study on Laser Shock Peening of Pure Al Correlating with Laser Shock Wave. Materials.

[23] Zabeen S., Preuss M., Withers P.J. (2015). Evolution of a Laser Shock Peened Residual Stress Field Locally with Foreign Object Damage and Subsequent Fatigue Crack Growth. Acta Mater..

[24] Chi J., Cai Z., Wan Z., Zhang H., Chen Z., Li L., Li Y., Peng P., Guo W. (2020). Effects of Heat Treatment Combined with Laser Shock Peening on Wire and Arc Additive Manufactured Ti17 Titanium Alloy: Microstructures, Residual Stress and Mechanical Properties. Surf. Coat. Technol..

[25] Bai Y., Wang H., Wang S., Huang Y., Chen Y., Zhang W., Ostendorf A., Zhou X. (2021). Life Cycle Strengthening of High-Strength Steels by Nanosecond Laser Shock. Appl. Surf. Sci..

[26] Wang C.Y., Cheng W., Shao Y.K., Luo K.Y., Lu J.Z. (2021). Cavitation Erosion Behaviour of AISI 420 Stainless Steel Subjected to Laser Shock Peening as a Function of the Coverage Layer in Distilled Water and Water-Particle Solutions. Wear.

[27] Tian Y., Liu Z., Li X., Zhang L., Li R., Jiang R., Dong F. (2018). The Cavitation Erosion of Ultrasonic Sonotrode during Large-Scale Metallic Casting: Experiment and Simulation. Ultrason. Sonochem..

[28] Yang H., Zhu Y., Zhang Y., Zhang X., Zuo L., Yin Y., Pei S. (2022). Investigation on Surface “Residual Stress Hole” of Thin Plate Subjected to Two Sided Laser Shock Processing. Opt. Laser Technol..

[29] Zhang X., Huang Z., Chen B., Zhang Y., Tong J., Fang G., Duan S. (2019). Investigation on Residual Stress Distribution in Thin Plate Subjected to Two Sided Laser Shock Processing. Opt. Laser Technol..

[30] McGinn P., Tretola G., Vogiatzaki K. (2022). Unified Modeling of Cavitating Sprays Using a Three-Component Volume of Fluid Method Accounting for Phase Change and Phase Miscibility. Phys. Fluids.

[31] Asano Y., Watanabe H., Noguchi H. (2020). Effects of Cavitation on Kármán Vortex behind Circular-Cylinder Arrays: A Molecular Dynamics Study. J. Chem. Phys..

[32] Qin Z., Li X., Xia D.H., Zhang Y., Feng C., Wu Z., Hu W. (2022). Effect of Compressive Stress on Cavitation Erosion-Corrosion Behavior of Nickel-Aluminum Bronze Alloy. Ultrason. Sonochem..

[33] Ouyang P., Luo X., Dong Z., Zhang S. (2022). Numerical Prediction of the Effect of Laser Shock Peening on Residual Stress and Fatigue Life of Ti-6Al-4V Titanium Alloy. Materials.

[34] He Z., Li C., Zhao S., Cui B., Li D., Yu H., Chen L., Fu T. (2019). Mathematical Model and Verification of Residual Stress Induced by Water Jet Peening. Metals.

[35] Hsu C.Y., Liang C.C., Teng T.L., Nguyen A.T. (2013). A Numerical Study on High-Speed Water Jet Impact. Ocean Eng..

[36] Rajesh N., Ramesh Babu N. (2006). Multidroplet Impact Model for Prediction of Residual Stresses in Water Jet Peening of Materials. Mater. Manuf. Process..

[37] Philipp A., Lauterborn W. (2000). Cavitation Erosion by Single Laser-Produced Bubbles. J. Fluid Mech..

[38] Brujan E.A., Matsumoto Y. (2012). Collapse of Micrometer-Sized Cavitation Bubbles near a Rigid Boundary. Microfluid. Nanofluid..

[39] Plesset M.S., Chapman R.B. (1970). Chapman Collapse of an Initially Spherical Vapour Cavity in the Neighbourhood of a Solid Boundary. J. Fluid Mech..

[40] Yang X., Li W., Fu Y., Ye Q., Xu Y., Dong X., Hu K., Zou Y. (2019). Finite Element Modelling for Temperature, Stresses and Strains Calculation in Linear Friction Welding of TB9 Titanium Alloy. J. Mater. Res. Technol..

[41] Roth C.C., Mohr D. (2014). Effect of Strain Rate on Ductile Fracture Initiation in Advanced High Strength Steel Sheets: Experiments and Modeling. Int. J. Plast..

[42] Rooze J., Rebrov E.V., Schouten J.C., Keurentjes J.T. (2013). Dissolved Gas and Ultrasonic Cavitation—A Review. Ultrason. Sonochem..

[43] Design of High-Entropy Alloy Coating for Cavitation Erosion Resistance by Different Energy-Induced Dynamic Cyclic Behaviors| ACS Applied Materials & Interfaces. https://pubs.acs.org/doi/10.1021/acsami.2c19210.

[44] Song S., Yang H., Su C., Jiang Z., Lu Z. (2016). Ultrasonic-Microwave Assisted Synthesis of Stable Reduced Graphene Oxide Modified Melamine Foam with Superhydrophobicity and High Oil Adsorption Capacities. Chem. Eng. J..

[45] Mitelea I., Bordeaşu I., Pelle M., Crăciunescu C. (2015). Ultrasonic Cavitation Erosion of Nodular Cast Iron with Ferrite-Pearlite Microstructure. Ultrason. Sonochem..

[46] Nair R.B., Arora H.S., Mukherjee S., Singh S., Singh H., Grewal H.S. (2018). Exceptionally High Cavitation Erosion and Corrosion Resistance of a High Entropy Alloy. Ultrason. Sonochem..

[47] Rajput A., Ramkumar J., Mondal K. (2021). Effect of Pearlitic Morphology with Varying Fineness on the Cavitation Erosion Behavior of Eutectoid Rail Steel. Ultrason. Sonochem..

[48] Hanke S., Beyer M., Silvonen A., dos Santos J.F., Fischer A. (2013). Cavitation Erosion of Cr60Ni40 Coatings Generated by Friction Surfacing. Wear.

